# Supplementary material for: Broadly Antiviral Activities of TAP1 through Activating the TBK1-IRF3-Mediated Type I Interferon Production
Source: Int J Mol Sci. 2021 Apr 28;22(9):4668. doi: 10.3390/ijms22094668 (PMC8125511; doi:10.3390/ijms22094668)
Supplement: Supplementary file 1 [file ijms-22-04668-s001.zip › ijms-1113885-supplementary.pdf]

International Journal of *Molecular Sciences*

MDPI

Supplementary Material

Jin Zhao [1,2,†], Ruiting Li [1,2,†], Yanjun Li [1,2], Jiaoshan Chen [1,2], Fengling Feng [1,2] and Caijun Sun [1,2,*]

1 School of Public Health (Shenzhen), Sun Yat-sen University, Guangzhou 514400, China; zhaoj47@mail2.sysu.edu.cn (J.Z.); lirt9@mail2.sysu.edu.cn (R.L.); liyj253@mail2.sysu.edu.cn (Y.L.); chenjsh59@mail2.sysu.edu.cn (J.C.); fengfling@mail.sysu.edu.cn (F.F.); suncaijun@mail.sysu.edu.cn (C.S.)
2 Key Laboratory of Tropical Disease Control (Sun Yat-sen University), Ministry of Education, Guangzhou 514400, China
* Correspondence: suncaijun@mail.sysu.edu.cn; Tel.: +86-20-83226383
† These authors contributed equally to this work.

Supplemental figures

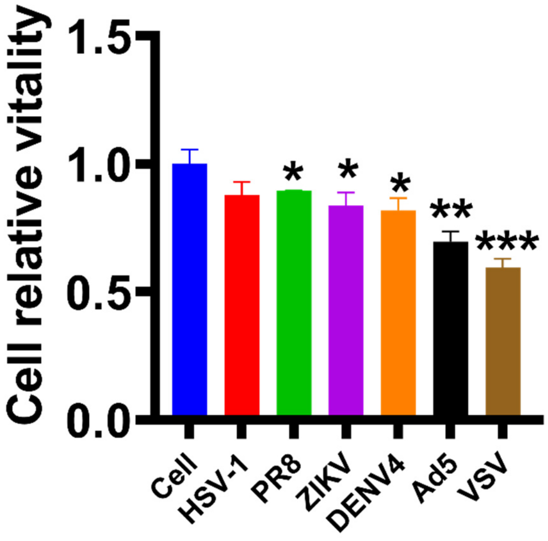

Figure S1. Detection of cell viability after TAP1 overexpression and infection with different viruses. HEK293T cells were infected by HSV-1, PR8, ZIKV, DENV4, Ad5, VSV infection (MOI=1) for 8 h respectively, and cell viability was measured by CCK8 kits. *$P < 0.05$, **$P < 0.01$, ***$P < 0.001$.

Citation: Zhao, J.; Li, R.; Li, Y.; Chen, J.; Feng, F.; Sun, C. Broadly Antiviral Activities of TAP1 through Activating the TBK1-IRF3 -Mediated Type I Interferon Production. *Int. J. Mol. Sci.* **2021**, *22*, 4668. https://doi.org/10.3390/ijms22094668

Academic Editor: Maria Grazia Romanelli and Jeffrey L. Platt

Received: 01 February 2021
Accepted: 25 April 2021
Published: 28 April 2021

Publisher's Note: MDPI stays neutral with regard to jurisdictional claims in published maps and institutional affiliations.

Copyright: © 2021 by the authors. Licensee MDPI, Basel, Switzerland. This article is an open access article distributed under the terms and conditions of the Creative Commons Attribution (CC BY) license (http://creativecommons.org/licenses/by/4.0/).

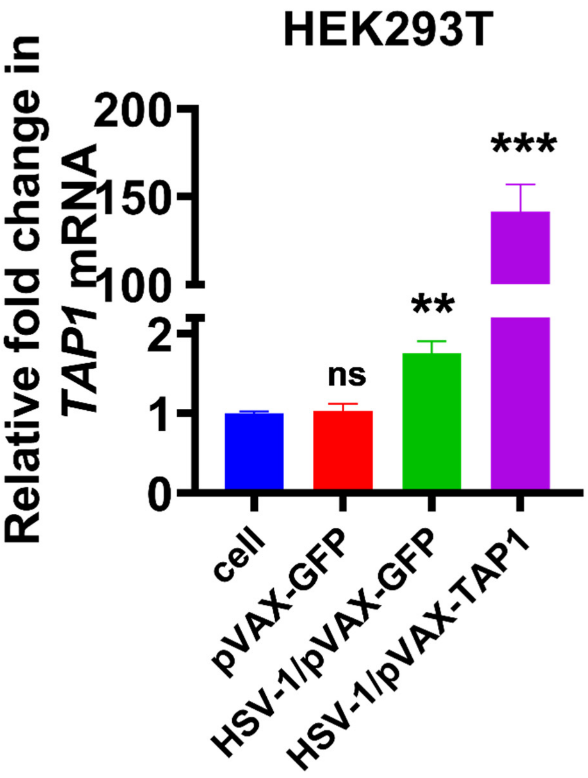

Figure S2. Detection of the level of TAP1 overexpression under uninfected HEK293T, pVAX-GFP(0.5μg/mL)-transfected HEK293T, HSV-1-infected pVAX-GFP-transfected and the HSV-1-infected and TAP1 (0.5μg/mL)-trasnfected HEK293T. The expression level of mRNA was normalized to the expression of β-actin, and the data from at least triplicates were shown as the mean ± SD. *$P < 0.05$, **$P < 0.01$, ***$P < 0.001$.

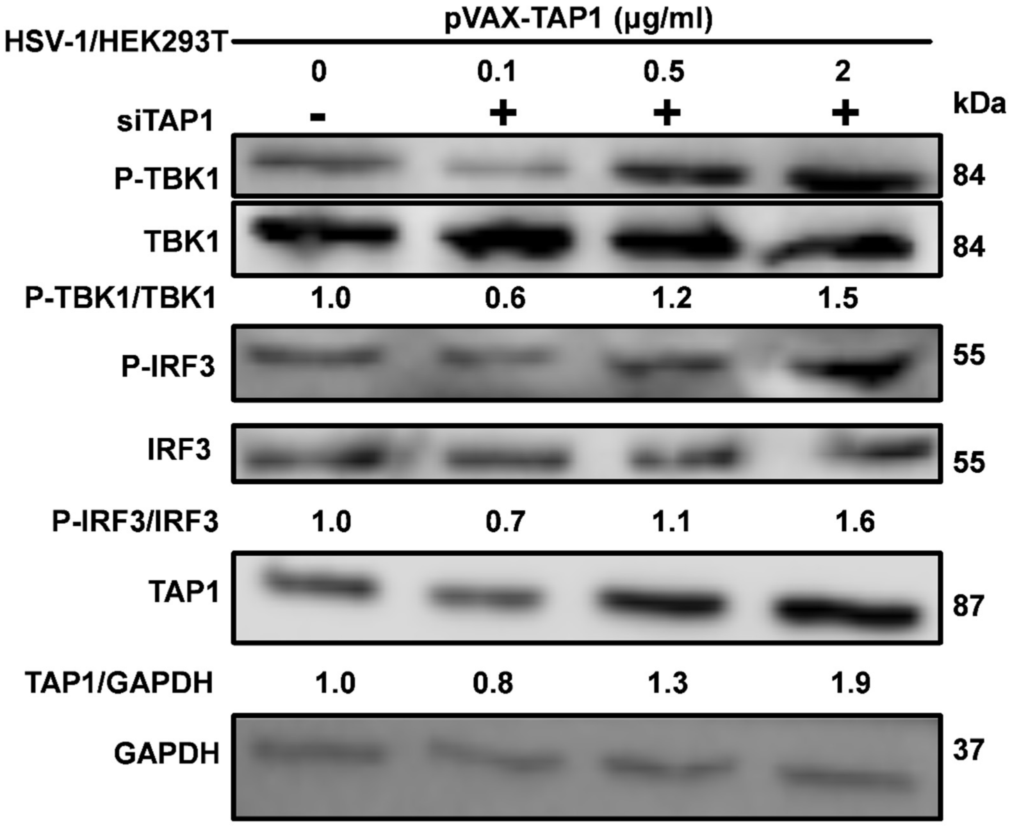

Figure S3. HEK293T cells were transfected with TAP1-expressing plasmid with different concentrations (0.1μg/mL, 0.5μg/mL, 2μg/mL), when transfecting siTAP1 with the same concentration as before for 24 h, followed by HSV-1 infection (MOI=0.25) for 8 h. Then, the expression of targeted proteins was detected by Western blot. The relative ratios of targeted protein and GAPDH were marked at the bottom of the pictures.
